# Lupus Retinopathy in Systemic Lupus Erythematosus: Prevalence, Phenotypic Spectrum, and Associations with Severe Disease Activity and Antiphospholipid Antibody Status

**DOI:** 10.3390/jcm15218697

**Published:** 2026-11-07

**Authors:** Shifat Shafin, Md Nazrul Islam, Mohammad Abul Kalam Azad, Abul Khair Ahmedullah, Manik Kumar Talukder, Tariq Reza Ali, Khondokar Mahbub-Uz-Zaman, Nira Ferdous

**Affiliations:** 1 Department of Rheumatology, Bangladesh Medical University, Dhaka 1000, Bangladesh; durgapur@uhfpo.dghs.gov.bd (S.S.); nazrul1islam@bsmmu.edu.bd (M.N.I.); dr.ahmedullah@bsmmu.edu.bd (A.K.A.); 2 Department of Pediatric Rheumatology, Bangladesh Medical University, Dhaka 1000, Bangladesh; talukder.m@bsmmu.edu.bd; 3 Department of Ophthalmology, Bangladesh Medical University, Dhaka 1000, Bangladesh; tariqrezaali@bmu.ac.bd; 4 Combined Military Hospital, Dhaka 1206, Bangladesh; cmhdkj@gmail.com; 5 Department of Medicine, MH Samorita Medical College, Dhaka 1208, Bangladesh; info@mhsamorita.edu.bd

**Keywords:** systemic lupus erythematosus, lupus retinopathy, antiphospholipid antibodies, disease activity, retinal vasculitis

## Abstract

**Background/Objectives**: Lupus retinopathy is an uncommon but clinically important manifestation of systemic lupus erythematosus (SLE) that may reflect systemic vascular injury and increased activity. However, data from South Asian populations are limited. To determine the prevalence, clinical spectrum, and factors associated with lupus retinopathy in patients with SLE, with particular emphasis on disease activity and antiphospholipid antibody (aPL) status. **Methods**: This hospital-based cross-sectional study included adults with SLE who met the 2019 European Alliance of Associations for Rheumatology/American College of Rheumatology classification criteria and were consecutively recruited at a tertiary referral center in Bangladesh. All participants underwent comprehensive ophthalmological examinations, including dilated fundus examinations and fundus photography. Optical coherence tomography (OCT) and fundus fluorescein angiography (FFA) were conducted as needed in the study. We evaluated disease activity using the SLE Disease Activity Index 2000 (SLEDAI-2K). We used logistic regression to examine the relationship between lupus retinopathy and other factors. We also analyzed the association between aPL status and retinopathy phenotype using Fisher’s exact test. **Results**: Among 159 patients with SLE, 18 (11.3%) had ocular involvement and 16 (10.1%) had lupus retinopathy. Of the 16 patients with retinopathy, 11 (68.8%) had mild, 2 (12.5%) had moderate, and 3 (18.8%) had severe disease; 3 (18.8%) had occlusive and 13 (81.3%) had non-occlusive; retinonocclusive systemic disease activity was independently associated with lupus retinopathy (adjusted odds ratio [aOR], 7.20; 95% confidence interval [CI], 1.78–29.11; *p* = 0.006). Renal involvement was not independently associated with retinopathy (aOR, 2.31; 95% CI, 0.56–9.58; *p* = 0.247). Among patients with available aPL results, aPL positivity was not significantly associated with lupus retinopathy (41.7% vs. 26.7%; *p* = 0.315). However, among the 12 patients with retinopathy who had available aPL results, occlusive retinopathy occurred in 3/5 (60.0%) aPL-positive patients compared with 0/7 (0%) aPL-negative patients (Fisher’s exact test, *p* = 0.045). OCT and FFA performed in eight patients demonstrated macular edema and bilateral retinal capillary non-perfusion in all cases. **Conclusions**: Lupus retinopathy affected approximately one in ten patients with SLE in this Bangladeshi cohort and was strongly associated with severe systemic disease. Although aPL positivity was not associated with retinopathy, it was associated with the occlusive phenotype in an exploratory analysis. These findings warrant confirmation in larger prospective studies with systematic aPL testing.

## 1. Introduction

Systemic lupus erythematosus (SLE) is a chronic autoimmune disease characterized by multisystem involvement and a relapsing–remitting clinical course [1,2,3]. Its manifestations may involve multiple organ systems, including the eye, where virtually any ocular structure may be affected [1,2,3]. Common ophthalmic manifestations include keratoconjunctivitis sicca, iridocyclitis, retinal vasculitis, vaso-occlusive retinopathy, choroidopathy, and optic neuropathy [1,2,3].

Among these manifestations, lupus retinopathy is clinically important because it can threaten vision and may be associated with systemic disease activity and vascular involvement [4,5,6]. The reported prevalence of lupus retinopathy varies across studies and populations and appears to be influenced by disease activity and the characteristics of the population studied [4,5,6]. Lupus retinopathy has been associated with increased disease activity and major organ involvement, suggesting that retinal abnormalities may serve as clinical markers of systemic disease severity [4,5,6]. Clinically, lupus retinopathy encompasses a spectrum of retinal abnormalities, from mild microangiopathic changes, such as cotton wool spots and retinal hemorrhages, to severe vaso-occlusive disease that may cause irreversible visual loss [1,2,3,4,5,6].

Lupus retinopathy is multifactorial and may involve immune complex-mediated microangiopathy, complement activation, endothelial dysfunction, and inflammatory vascular injury, resulting in retinal microvascular abnormalities and ischemia [3,4,5,6]. Thrombotic mechanisms may also contribute, particularly in patients with antiphospholipid antibodies (aPL), which are associated with retinal vascular occlusive manifestations [2,3,5]. However, the relative contributions of inflammatory and thrombotic mechanisms may vary among patients.

Advances in retinal imaging, including optical coherence tomography (OCT), fundus fluorescein angiography (FFA), and optical coherence tomography angiography (OCTA), have improved the detection and characterization of retinal involvement in SLE, including structural and microvascular abnormalities that may not be apparent on routine fundus examinations [7,8,9]. These modalities provide complementary information on retinal structural abnormalities, macular involvement, vascular leakage, and areas of capillary non-perfusion [7,8,9].

Although lupus retinopathy has been studied in several populations [6,7,8,9], data from South Asia, particularly Bangladesh, remain limited. Consequently, the prevalence, clinical spectrum, and factors associated with lupus retinopathy in Bangladeshi patients with SLE remain insufficiently characterized. Furthermore, retinal involvement may be asymptomatic in its early stages and may, therefore, remain undetected without ophthalmologic assessment [4,6,7,8,9]. A recent Bangladeshi case series describing the severe visual consequences of retinal vasculitis in patients with SLE further highlights the clinical importance of ophthalmological evaluation in this population [10].

A better understanding of the clinical characteristics of lupus retinopathy and their associations with disease activity, renal involvement, and aPL status may help identify patients at a higher risk of retinal involvement and support appropriate multidisciplinary evaluation [5,6]. Therefore, this study aimed to determine the prevalence and clinical spectrum of lupus retinopathy and identify the clinical and laboratory factors associated with its occurrence, with particular emphasis on disease activity, renal involvement, and aPL status, among Bangladeshi patients with SLE.

## 2. Methods

### 2.1. Study Design and Setting

This hospital-based cross-sectional study was conducted in the Departments of Rheumatology and Ophthalmology at Bangladesh Medical University (BMU), formerly Bangabandhu Sheikh Mujib Medical University (BSMMU), Dhaka, Bangladesh, from 17 September 2023 to 17 September 2024. This study was reported in accordance with the Strengthening the Reporting of Observational Studies in Epidemiology (STROBE) guidelines [11].

We evaluated 186 consecutive patients with suspected or confirmed systemic lupus erythematosus (SLE) for eligibility. After applying predefined inclusion and exclusion criteria, we excluded 27 patients, leaving 159 eligible participants for the final analysis (see Figure 1).

### 2.2. Participants, Inclusion and Exclusion Criteria

Adults aged ≥18 years who fulfilled the 2019 European Alliance of Associations for Rheumatology/American College of Rheumatology (EULAR/ACR) classification criteria for systemic lupus erythematosus (SLE), including the obligatory entry criterion of a positive antinuclear antibody (ANA) test at a titer of ≥1:80, were eligible for inclusion [12]. Participants were consecutively recruited from the rheumatology inpatient and outpatient services during the study period.

Patients were excluded if they had an overlapping connective tissue disease, hypertension, recent ocular surgery that could interfere with retinal assessment, pregnancy, or were unable or unwilling to provide written informed consent or complete the required ophthalmological assessment. In accordance with a predefined study protocol, patients receiving antimalarial drugs or systemic corticosteroids and those with rapidly progressive glomerulonephritis (RPGN) were also excluded.

### 2.3. Sample Size Calculation

The sample size was calculated using the single-population proportion formula, assuming an expected prevalence of lupus retinopathy of 10.3%, based on Seth et al. [13]. With a 95% confidence level and 5% absolute precision, the minimum required sample size was 142 participants [14]. After allowing for approximately 10% non-evaluable participants or incomplete assessments, the target sample size was 158 participants. Ultimately, this study included 159 eligible participants.

### 2.4. Data Collection

Data were collected using a standardised case report form. Demographic and clinical information, including age, sex, residence, education, marital status, age at disease onset, disease duration, clinical manifestations, medication history, and comorbidities, was obtained through structured interviews, physical examinations, and review of medical records.

Laboratory investigations included complete blood count, erythrocyte sedimentation rate (ESR), C-reactive protein (CRP), serum creatinine, routine urinalysis, and assessment of proteinuria using the urine protein-to-creatinine ratio or, when available, 24-h urinary protein estimation.

Immunological investigations included antinuclear antibody (ANA), anti-double-stranded DNA (anti-dsDNA) antibodies, complement C3 and C4 levels, direct Coombs test, and antiphospholipid antibody (aPL) testing. aPL testing included lupus anticoagulant (LA), anticardiolipin (aCL) IgG and IgM, and anti-β_2_-glycoprotein I (anti-β_2_GPI) IgG and IgM antibodies. aPL testing was performed when clinically indicated and was therefore not available for all participants.

Disease activity was assessed using the Systemic Lupus Erythematosus Disease Activity Index 2000 (SLEDAI-2K) [15]. Health-related quality of life was assessed using the 12-Item Short Form Health Survey (SF-12) [16], and cumulative organ damage was assessed using the Systemic Lupus International Collaborating Clinics/American College of Rheumatology Damage Index (SLICC/ACR DI) [17].

### 2.5. Assessment of Disease Activity

Disease activity was categorised according to the SLEDAI-2K score as inactive (score 0), mild (1–5), moderate (6–10), or severe (>10) [15]. For regression analysis, we defined severe disease activity a priori as a SLEDAI-2K score > 10.

### 2.6. Ophthalmologic Assessment

All participants underwent a standardised comprehensive ophthalmological examination by experienced ophthalmologists, irrespective of the presence or absence of visual symptoms. The assessment included best-corrected visual acuity, intraocular pressure measurement, slit-lamp examination, dilated fundus examination, indirect ophthalmoscopy (HEINE indirect ophthalmoscope (HEINE Optotechnik GmbH & Co. KG, Gilching, Germany)), and digital colour fundus photography.

The retinal assessment included the evaluation of cotton-wool spots, retinal haemorrhages, hard exudates, retinal vasculitis, vascular attenuation, retinal arterial or venous occlusion, retinal ischemia, neovascularisation, and vitreous haemorrhage.

Optical coherence tomography (OCT) and fundus fluorescein angiography (FFA) were performed when clinically indicated and were not mandatory screening investigations. OCT (NIDEK OCT system (NIDEK Co., Ltd., Gamagori, Aichi, Japan)) was used to evaluate suspected macular or other structural abnormalities, whereas FFA (Canon system (Canon Medical Systems Corporation, Otawara, Tochigi, Japan)) was performed when retinal vascular abnormalities, ischemia, or vascular leakage were suspected. Experienced ophthalmologists reviewed the retinal photographs and imaging findings using standardised clinical criteria [18,19].

### 2.7. Definition of Lupus Retinopathy

The primary outcome was lupus retinopathy, defined as retinal abnormalities attributable to SLE after excluding other plausible causes. We based the diagnosis primarily on clinical ophthalmologic findings, using OCT and/or FFA as supportive investigations when clinically indicated.

### 2.8. Classification of Retinopathy Severity

We classified lupus retinopathy severity based on the most severe retinal abnormality identified in either eye.

Mild: cotton-wool spots, retinal hemorrhages, and/or hard exudates.

Moderate: Retinal vasculitis and/or vascular attenuation.

Severe: Retinal arterial or venous occlusion, neovascularization, and/or vitreous hemorrhage.

We predefined this classification for the present study and adapted it from previously described clinical classifications of lupus retinopathy [13,19,20,21,22].

### 2.9. Classification of Retinopathy Phenotype

We classified lupus retinopathy as occlusive or nonocclusive. Nonocclusive retinopathy was defined as retinal abnormalities without evidence of retinal arterial or venous occlusion, whereas occlusive retinopathy was defined as documented retinal arterial or venous occlusion.

### 2.10. Definition of Renal Involvement

We defined renal involvement as lupus nephritis diagnosed by the treating rheumatologist or nephrologist based on compatible clinical, laboratory, and/or renal biopsy findings documented in the medical records.

### 2.11. Antiphospholipid Antibody Assessment

aPL positivity was defined as a positive result for at least one of the following: LA, aCL IgG or IgM, or anti-β_2_GPI IgG or IgM.

Among the 159 participants, aPL results were available for 98 (61.6%) patients. Testing followed routine clinical practice and clinical indications rather than being performed systematically in all the participants. Among those tested, 28/98 (28.6%) were positive for at least one aPL antibody. Individual antibody categories were not mutually exclusive because participants could have more than one positive antibody.

For analyses examining the association between aPL status and retinopathy phenotype, we included only the 12 patients with lupus retinopathy with available aPL results. Because aPL testing was not systematic and the subgroup was small, we considered these analyses exploratory and interpreted them with caution.

### 2.12. Bias and Quality Assurance

We consecutively recruited eligible participants to minimize selection bias. We used standardized protocols for clinical, laboratory, and ophthalmological assessments to reduce measurement variability. We cross-checked clinical and laboratory information against medical records and reviewed completed case report forms for completeness and consistency before finalizing the database.

Because aPL testing was performed based on clinical indications rather than systematically in all participants, consider potential selection bias and confounding factors when interpreting aPL-related findings.

### 2.13. Missing Data

Missing data were minimal for most variables, although the availability of individual laboratory investigations varied because some tests were performed according to routine clinical care protocols. aPL results were available for 98 (61.6%) of the 159 participants. No imputations were performed. Analyses involving aPL status and other variables with missing observations were based on the available cases, with denominators reported where applicable.

### 2.14. Statistical Analysis

Statistical analyses were performed using IBM SPSS Statistics for Windows, version 25.0 (IBM Corp., Armonk, NY, USA). Continuous variables are presented as mean ± standard deviation (SD) or median (interquartile range [IQR]), as appropriate, and categorical variables as frequencies and percentages.

Patients with and without lupus retinopathy were compared using the independent-samples *t* test or Mann–Whitney U test for continuous variables, as appropriate, and the chi-square test or Fisher’s exact test for categorical variables, as appropriate.

Univariable binary logistic regression analysis was performed to identify factors associated with lupus retinopathy. Variables with *p* < 0.10 in the univariable analyses or those considered clinically relevant were included in the multivariable analysis. Given that only 16 participants had lupus retinopathy, the final multivariable model was deliberately restricted to two clinically relevant predictors—severe disease activity and renal involvement—to minimize the risk of model overfitting.

Results of the logistic regression analyses are presented as odds ratios (ORs) for univariable analyses and adjusted odds ratios (aORs) for multivariable analysis, with corresponding 95% confidence intervals (CIs). The association between aPL status and retinopathy phenotype was assessed using Fisher’s exact test because of the small cell counts. All statistical tests were two-sided, and *p* < 0.05 was considered statistically significant.

### 2.15. Ethical Considerations

The Institutional Review Board of Bangladesh Medical University (BMU), formerly Bangabandhu Sheikh Mujib Medical University (BSMMU), approved the study (Approval No. BSMMU/2023/12947) on 16 September 2023. Written informed consent was obtained from all participants before enrollment. Participants were informed about the study procedures, including the ophthalmological assessment, and participation was voluntary. The study was conducted in accordance with the Declaration of Helsinki [23].

### 2.16. Use of Artificial Intelligence

AI-assisted tools were used solely for language editing, grammar correction, and improving the manuscript readability. They were not used for the study design, statistical analysis, interpretation of results, or formulation of scientific conclusions. All AI-assisted content was reviewed and verified by the authors, who are responsible for the accuracy, integrity, and final content of the manuscript.

## 3. Results

**Baseline Characteristics:** Of the 186 patients assessed for eligibility, 27 were excluded according to the predefined criteria, leaving 159 patients with SLE for the final analysis (Figure 1). Of these, 149 (93.7%) were female, with a mean age of 26.2 ± 7.2 years. The mean age at disease onset was 23.1 ± 7.2 years, and the median disease duration was 24 months (IQR 6–53). Most participants were married (61.6%), lived in rural areas (63.5%), and had at least a secondary education (86.2%) (Table 1).

**Clinical and Laboratory Characteristics:** Table 2 summarises the clinical and laboratory characteristics of the patients. SLEDAI-2K disease activity was mild in 46 patients (28.9%), moderate in 57 (35.8%), and severe in 56 (35.2%). The mean SLICC/ACR Damage Index score was 0.38 ± 0.86. Mucocutaneous involvement was the most common (75.5%), followed by musculoskeletal (70.4%) and renal involvement (49.1%). The mean PCS-12 and MCS-12 scores were 34.8 ± 9.3 and 36.6 ± 8.7, respectively. The mean haemoglobin level was 10.1 ± 1.9 g/dL. Anti-dsDNA was positive in 92/129 (71.3%) patients, whereas low C3 and C4 levels were present in 90/122 (73.8%) and 48/122 (39.3%) patients, respectively. Among the 98 patients tested for aPL, 28 (28.6%) were positive.

**Ocular Manifestations and Lupus Retinopathy:** Ocular involvement occurred in 18/159 patients (11.3%), including lupus retinopathy in 16 (10.1%) (Table 3). Other ocular findings included anterior uveitis, blepharitis, and hordeolum (one patient each, 0.6%).

Among patients with lupus retinopathy, cotton-wool spots were the most common finding (8/16, 50.0%), followed by retinal vasculitis and hard exudates (5/16 each, 31.3%), optic disc edema (4/16, 25.0%), and retinal hemorrhage (3/16, 18.8%). The findings were not mutually exclusive (Table 3; Figure 2A). Most patients had mild retinopathy (11/16, 68.8%), followed by moderate (2/16, 12.5%) and severe retinopathy (3/16, 18.8%). Non-occlusive retinopathy was observed in 13 patients (81.3%), and occlusive retinopathy was observed in three (18.8%). Retinopathy was bilateral in 11 patients (68.8%) and unilateral in 5 (31.3%). Gradual visual loss was the most common presentation (10/16, 62.5%); 4 patients (25.0%) were asymptomatic, and 2 (12.5%) had sudden visual loss (Figure 2B).

**Comparison of Patients with and without Lupus Retinopathy:** Patients with lupus retinopathy had more severe disease activity (13/16 [81.3%] vs. 43/143 [30.1%], *p* < 0.001) and renal involvement (13/16 [81.3%] vs. 65/143 [45.5%] (*p* = 0.007) than those without retinopathy (Table 4). They also had lower PCS-12 scores (30.25 ± 6.01 vs. 35.30 ± 9.49, *p* = 0.039) and hemoglobin levels (9.23 ± 1.52 vs. 10.24 ± 1.91 g/dL, *p* = 0.045). The SLICC/ACR Damage Index was borderline higher in the retinopathy group (*p* = 0.050).

We observed no significant differences in other demographic, clinical, or laboratory variables, including anti-dsDNA, low C3, and aPL positivity. Low C4 levels were more frequent in the retinopathy group but were not statistically significant (60.0% vs. 36.4%, *p* = 0.080).

**Factors Associated with Lupus Retinopathy:** We performed univariable and multivariable binary logistic regression analyses to identify factors associated with lupus retinopathy (Table 5). In univariable analyses, severe disease activity (SLEDAI-2K > 10), renal involvement, higher ESR, lower hemoglobin concentration, and lower PCS-12 scores were significantly associated with lupus retinopathy. Low complement C4 levels also met the prespecified screening threshold (*p* < 0.10).

Because only 16 patients had lupus retinopathy, the final multivariable model included only two clinically relevant predictors—severe disease activity and renal involvement—to minimize the risk of overfitting. Severe disease activity remained independently associated with lupus retinopathy (adjusted odds ratio [aOR], 7.20; 95% CI, 1.78–29.11; *p* = 0.006), whereas renal involvement was not independently associated after adjustment (aOR, 2.31; 95% CI, 0.56–9.58; *p* = 0.247).

**Antiphospholipid Antibody Status and Retinopathy Phenotype:** Overall, aPL antibody positivity was not significantly associated with lupus retinopathy. Among participants with available aPL results, 5/12 patients with retinopathy (41.7%) and 23/86 without retinopathy (26.7%) were aPL positive (*p* = 0.315).

Among the 12 patients with retinopathy and available aPL results, five were aPL-positive, and seven were aPL-negative (Table 6). Occlusive retinopathy occurred in 3/5 (60.0%) compared with 0/7 (0.0%) in aPL-negative patients, whereas non-occlusive retinopathy occurred in 2/5 (40.0%) and 7/7 (100.0%) patients, respectively (Fisher’s exact test, *p* = 0.045). Because aPL testing was performed based on clinical indication rather than systematically, and the analysis included only 12 patients, this association should be considered exploratory and interpreted with caution. cautiously.

**Retinal Imaging Findings:** OCT and FFA were performed in eight of the 16 patients with lupus retinopathy because of clinically indicated retinal involvement (Table 6). FFA demonstrated bilateral retinal capillary non-perfusion in all 8 patients (100.0%), perivascular leakage in 6 (75.0%), and perivascular sheathing in 5 (62.5%). OCT demonstrated macular edema in all eight patients (100.0%), including cystoid macular edema in four (50.0%). The imaging findings were not mutually exclusive.

## 4. Discussion

This cross-sectional study evaluated the prevalence, clinical characteristics, and factors associated with lupus retinopathy in patients with SLE at a tertiary referral center in Bangladesh. We identified ocular involvement in 18 of 159 patients (11.3%), including 16 (10.1%) with lupus-related retinopathy. The prevalence of lupus retinopathy in our study was comparable to the ~10% reported by Seth et al., who also showed an association between lupus retinopathy and active systemic disease [13]. Our findings are consistent with previous studies showing associations between lupus retinopathy, increased systemic disease activity, and major organ involvement [4,5,6,13,20,24].

Cotton wool spots were the most frequent retinal finding (50.0%), followed by retinal vasculitis and hard exudates (31.3% each). These findings are consistent with the spectrum of retinal microvascular involvement in SLE. The pathogenesis is multifactorial and may involve immune complex-mediated vascular injury, complement activation, endothelial dysfunction, inflammation, and thrombosis [1,2,3,19,20,24]. Most patients had nonocclusive retinopathy (81.3%), whereas 18.8% had occlusive disease. Bilateral involvement occurred in 68.8% of patients, and four patients were asymptomatic, highlighting the possibility of clinically silent retinal involvement in patients with SLE.

Among the eight patients who underwent clinically indicated retinal imaging, FFA demonstrated bilateral capillary non-perfusion in all patients. At the same time, OCT showed macular edema in all 8, including cystoid macular edema in 4. These findings support the complementary value of multimodal retinal imaging in characterizing retinal vascular and macular abnormalities in selected patients with suspected retinal involvement [7,8,9,18,19]. However, because OCT and FFA were not performed systematically in all participants, these findings should not be considered representative of the entire study cohort.

Patients with lupus retinopathy had more severe disease activity and greater renal involvement, along with lower hemoglobin levels and PCS-12 scores than those without retinopathy. In the multivariable analysis, severe disease activity remained independently associated with lupus retinopathy (aOR 7.20, 95% confidence interval [CI], 1.78–29.11; *p* = 0.006), whereas renal involvement was not (aOR 2.31, 95% CI, 0.56–9.58; *p* = 0.247). These findings support the concept that lupus retinopathy may be associated with overall systemic disease activity rather than isolated organ involvement [4,5,6,13,20,24]. The lack of significant associations with anti-dsDNA antibodies or complement levels may reflect the small number of retinopathy events and the study’s limited statistical power.

The association between aPL status and the occlusive phenotype warrants further investigation. Among the 12 patients with retinopathy with available aPL results, occlusive retinopathy occurred in three of five aPL-positive patients, compared with none of the seven aPL-negative patients. This finding is biologically plausible, given the reported association between aPL and retinal vaso-occlusive disease [21,22,25]. However, aPL testing was not systematic, and the subgroup was very small; therefore, this finding should be considered exploratory and hypothesis-generating, rather than definitive. Larger prospective studies with systematic aPL testing are required to confirm this association.

These findings have potential clinical implications. As severe systemic disease activity was independently associated with lupus retinopathy and some affected patients were asymptomatic, ophthalmological assessment may provide useful additional information in patients with active or severe SLE. However, these findings do not establish whether routine ophthalmological screening of all patients with SLE improves clinical outcomes.

This study had several strengths, including consecutive recruitment, systematic ophthalmological assessment regardless of symptoms, standardized assessment of SLE disease activity, and collaboration between rheumatology and ophthalmology. Limitations include the single-center design, small number of retinopathy cases, incomplete and non-systematic aPL testing, missing laboratory data, and cross-sectional design, which limits causal inference. Furthermore, OCT and FFA were performed only based on clinical indications rather than systematically, which may have limited detection of subclinical retinal abnormalities in the study.

## 5. Conclusions

Lupus retinopathy was identified in approximately one in ten patients with SLE in this Bangladeshi cohort and was independently associated with severe systemic disease. Although aPL positivity was not significantly associated with retinopathy, its possible association with the occlusive phenotype warrants further investigation in larger prospective studies.

## Figures and Tables

**Figure 1:**
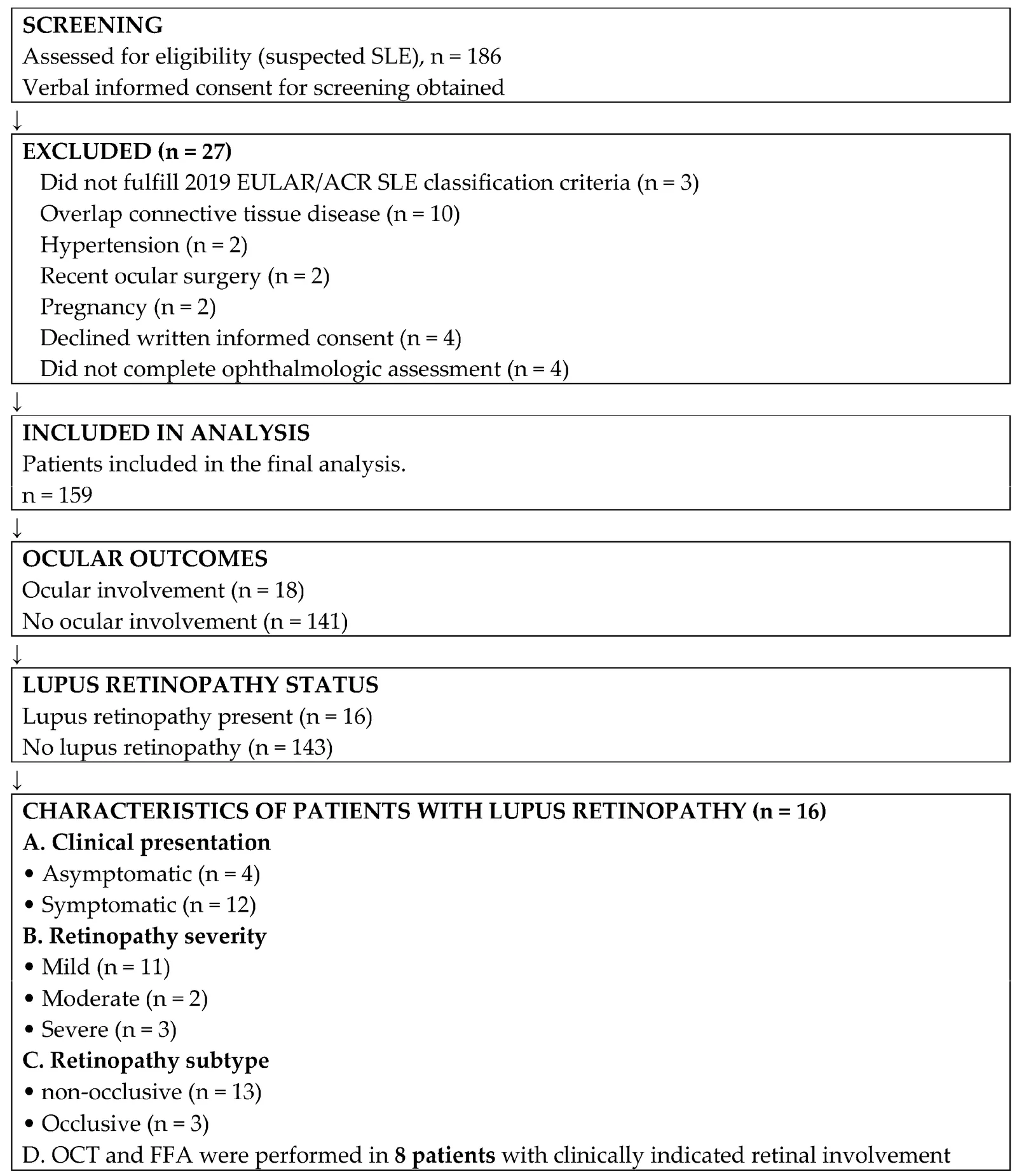
STROBE flow diagram illustrating participant selection and ophthalmological assessment. Of the 186 patients assessed for eligibility, 27 were excluded according to the predefined criteria, and 159 were included in the final analysis. Ocular involvement was observed in 18 patients, including 16 with retinopathy. OCT and FFA were performed in eight patients with clinically indicated retinal involvement.

**Figure 2:**
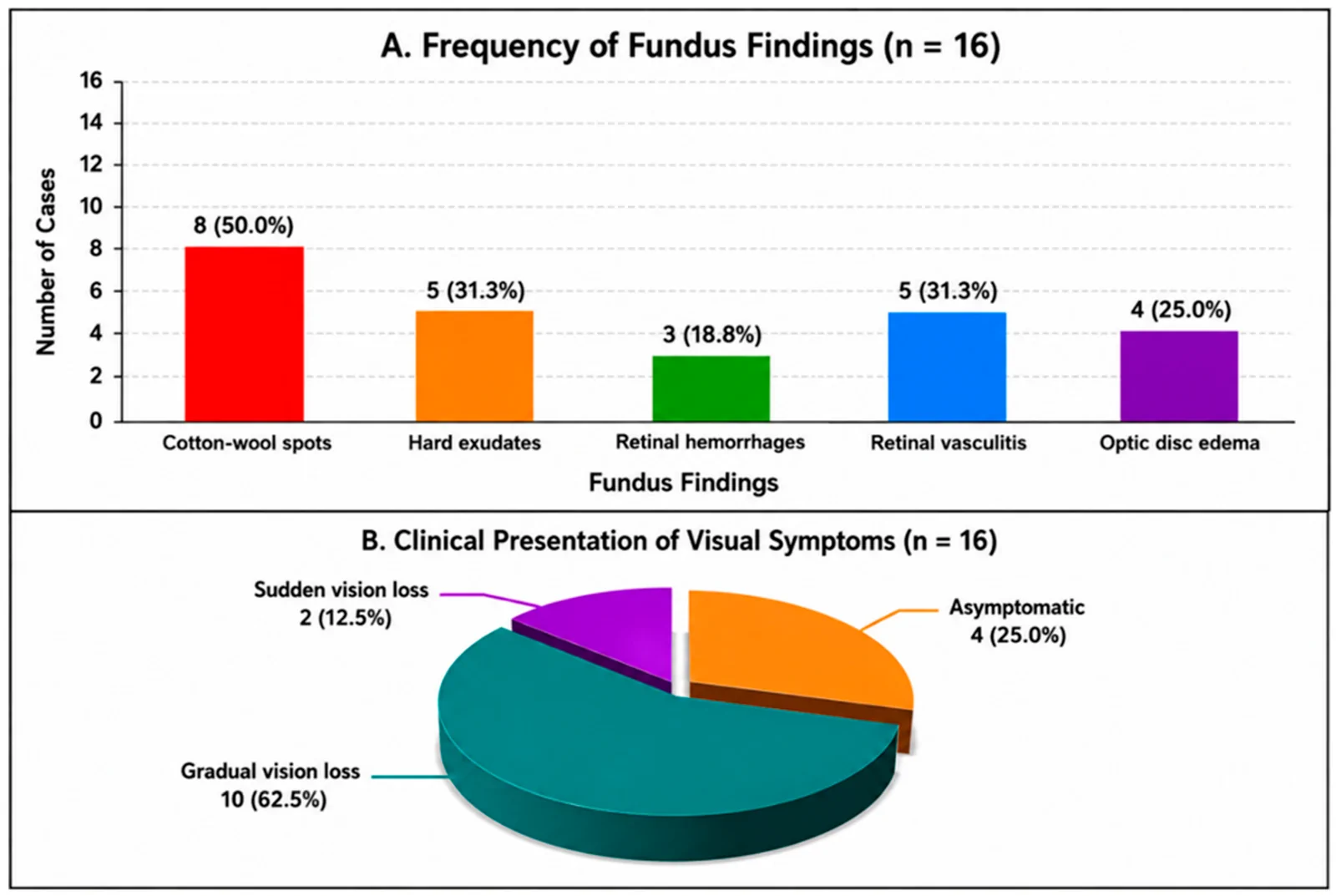
Clinical presentation and fundus findings in patients with lupus retinopathy (*n* = 16). Panel (**A**) shows the frequencies of fundus abnormalities. Cotton-wool spots were the most common finding, occurring in eight patients (50.0%), followed by hard exudates in five (31.3%), retinal vasculitis in five (31.3%), optic disc edema in four (25.0%), and retinal hemorrhages in three (18.8%). Patients may have more than one fundus abnormality; therefore, these findings were not mutually exclusive. Panel (**B**) shows the clinical presentation of the visual symptoms. Ten patients (62.5%) reported gradual vision loss, four (25.0%) were asymptomatic, and two (12.5%) had sudden vision loss. Patients could have more than one fundus abnormality; therefore, the fundus findings were not mutually exclusive, and percentages do not sum to 100%.

**Table 1:** Sociodemographic and disease-related characteristics of patients with systemic lupus erythematosus (*n* = 159).

Variable	Value
Age, years, mean ± SD	26.2 ± 7.2
Female sex, *n* (%)	149 (93.7)
Married, *n* (%)	98 (61.6)
Rural residence, *n* (%)	101 (63.5)
Education level ≥ secondary, *n* (%)	137 (86.2)
Age at disease onset, years, mean ± SD	23.1 ± 7.2
Disease duration, months, median (IQR)	24 (6–53)

**Legend:** Data are presented as mean ± SD, median (IQR), or *n* (%), as appropriate. We calculated the disease duration from the date of SLE diagnosis to the date of study enrollment. **Abbreviations:** IQR, interquartile range; SD, standard deviation; SLE, systemic lupus erythematosus.

**Table 2:** Clinical and laboratory characteristics of patients with systemic lupus erythematosus (*n* = 159).

Variable	Value
**Body mass index category, *n* (%)**	
Underweight	29/158 (18.4)
Normal weight	65/158 (41.1)
Overweight	32/158 (20.3)
Obese	32/158 (20.3)
**Disease characteristics**	
**SLEDAI-2K disease activity, *n* (%)**	
Mild	46 (28.9)
Moderate	57 (35.8)
Severe	56 (35.2)
SLICC/ACR Damage Index, mean ± SD	0.38 ± 0.86
PCS-12 score, mean ± SD	34.8 ± 9.3
MCS-12 score, mean ± SD	36.6 ± 8.7
**Organ involvement, *n* (%)**	
Mucocutaneous	120 (75.5)
Musculoskeletal	112 (70.4)
Renal	78 (49.1)
Hematological	40 (25.2)
Neuropsychiatric	28 (17.6)
**Laboratory findings**	
Hemoglobin, g/dL, mean ± SD	10.1 ± 1.9
Leukopenia, *n* (%)	3 (1.9)
Thrombocytopenia, *n* (%)	16 (10.1)
Direct Coombs test positive †, *n*/N (%)	20/87 (23.0)
Anti-dsDNA positive †, *n*/N (%)	92/129 (71.3)
Low complement C3 †, *n*/N (%)	90/122 (73.8)
Low complement C4 †, *n*/N (%)	48/122 (39.3)
Antiphospholipid antibody positive †, *n*/N (%)	28/98 (28.6)

Data are presented as mean ± SD, *n* (%), or *n*/N (%), as appropriate. Body mass index data were available for 158 participants. We classified BMI according to the standard World Health Organization criteria as underweight (<18.5 kg/m^2^), normal weight (18.5–24.9 kg/m^2^), overweight (25.0–29.9 kg/m^2^), and obese (≥30.0 kg/m^2^). Organ involvement categories were not mutually exclusive; therefore, the percentages do not sum to 100%. Variables marked with † were calculated using participants with available data (denominators shown as *n*/N). Antiphospholipid antibody positivity was defined as positivity for one or more of the following: lupus anticoagulant (LA), anticardiolipin antibody (aCL) IgG or IgM, and anti-β_2_-glycoprotein I antibody (anti-β_2_GPI) IgG or IgM. Antiphospholipid antibody testing was performed based on clinical indications and was not systematic. **Abbreviations:** aCL, anticardiolipin antibody; anti-β_2_GPI, anti-β_2_-glycoprotein I antibody; anti-dsDNA, anti-double-stranded DNA; BMI, body mass index; C3, complement component 3; C4, complement component 4; LA, lupus anticoagulant; MCS-12, Mental Component Summary of the 12-Item Short Form Health Survey; PCS-12, Physical Component Summary of the 12-Item Short Form Health Survey; SD, standard deviation; SLEDAI-2K, Systemic Lupus Erythematosus Disease Activity Index 2000; SLICC/ACR, Systemic Lupus International Collaborating Clinics/American College of Rheumatology.

**Table 3:** Ocular manifestations, retinal findings, and clinical characteristics of lupus retinopathy in patients with systemic lupus erythematosus (*n* = 159).

**A. Ocular manifestations in patients with systemic lupus erythematosus (*n* = 159)**	
**Variable**	***n* (%)**
Any ocular involvement	18 (11.3)
Lupus retinopathy	16 (10.1)
Anterior uveitis	1 (0.6)
Blepharitis	1 (0.6)
Hordeolum	1 (0.6)
**B. Retinal findings in patients with lupus retinopathy (*n* = 16)**	
**Retinal findings**	***n* (%)**
Cotton-wool spots	8 (50.0)
Retinal vasculitis	5 (31.3)
Hard exudates	5 (31.3)
Optic disc edema†	4 (25.0)
Retinal hemorrhages	3 (18.8)
**C. Clinical characteristics of lupus retinopathy (*n* = 16)**	
**Characteristic**	***n* (%)**
**Severity**	
Mild	11 (68.8)
Moderate	2 (12.5)
Severe	3 (18.8)
**Retinopathy type**	
Non-occlusive	13 (81.3)
Occlusive	3 (18.8)
**Laterality**	
Bilateral	11 (68.8)
Unilateral	5 (31.3)

**Legend:** Data are presented as *n* (%). The percentages in Panel A were calculated using the total study population (*n* = 159), whereas the percentages in Panels B and C were calculated using patients with lupus retinopathy (*n* = 16). Patients could have more than one fundus abnormality; therefore, the fundus findings in Panel B were not mutually exclusive, and the percentages do not sum to 100%.

**Table 4:** Comparison of demographic, clinical, and laboratory characteristics between patients with and without lupus retinopathy (*n* = 159).

Variable	No Retinopathy (*n* = 143)	Retinopathy (*n* = 16)	*p*-Value
**Demographic characteristics**			
Female sex, *n* (%)	134 (93.7)	15 (93.8)	1.000
Rural residence, *n* (%)	92 (64.3)	9 (56.3)	0.524
Age at disease onset, years, mean ± SD	23.28 ± 7.50	21.83 ± 4.15	0.451
Disease duration, months, mean ± SD	37.87 ± 45.71	32.62 ± 42.74	0.634
**Disease activity and patient-reported outcomes**			
Severe disease activity (SLEDAI-2K > 10), *n* (%)	43 (30.1)	13 (81.3)	<0.001
SLICC/ACR Damage Index, mean ± SD	0.35 ± 0.83	0.69 ± 1.08	0.050
PCS-12 score, mean ± SD	35.30 ± 9.49	30.25 ± 6.01	0.039
MCS-12 score, mean ± SD	36.66 ± 8.41	36.16 ± 10.98	0.828
**Organ involvement**			
Renal involvement, *n* (%)	65 (45.5)	13 (81.3)	0.007
Mucocutaneous involvement, *n* (%)	108 (75.5)	12 (75.0)	1.000
Musculoskeletal involvement, *n* (%)	100 (69.9)	12 (75.0)	0.780
Hematological involvement, *n* (%)	37 (25.9)	3 (18.8)	0.763
Neuropsychiatric involvement, *n* (%)	23 (16.1)	5 (31.3)	0.162
**Laboratory findings**			
Hemoglobin, g/dL, mean ± SD	10.24 ± 1.91	9.23 ± 1.52	0.045
ESR, mm/hour, mean ± SD	45.37 ± 27.67	69.69 ± 46.13	0.109
CRP, mg/L, mean ± SD †	35.58 ± 55.67	59.29 ± 67.43	0.089
Leukopenia, *n* (%)	3 (2.1)	0 (0.0)	1.000
Thrombocytopenia, *n* (%)	14 (9.8)	2 (12.5)	0.665
Anti-dsDNA positive ‡, *n*/N (%)	80/115 (69.6)	12/14 (85.7)	0.348
Low complement C3 ‡, *n*/N (%)	78/107 (72.9)	12/15 (80.0)	0.757
Low complement C4 ‡, *n*/N (%)	39/107 (36.4)	9/15 (60.0)	0.080
Antiphospholipid antibody positive ‡, *n*/N (%)	23/86 (26.7)	5/12 (41.7)	0.315
Direct Coombs test positive ‡, *n*/N (%)	18/76 (23.7)	2/11 (18.2)	1.000

Data are presented as mean ± SD, *n* (%), or *n*/N (%), as appropriate. We compared continuous variables using the independent-samples *t*-test or the Mann–Whitney U test, as appropriate. We compared categorical variables using the chi-square or Fisher’s exact test, as appropriate. † CRP data were available for 70 participants (62 without retinopathy and eight with retinopathy) and were compared using the Mann–Whitney U test because of non-normal distribution. ‡ Percentages were calculated among participants with available laboratory data; denominators are provided as *n*/N. **Abbreviations:** anti-dsDNA, anti-double-stranded DNA; C3, complement component 3; C4, complement component 4; CRP, C-reactive protein; ESR, erythrocyte sedimentation rate; MCS-12, Mental Component Summary of the 12-Item Short Form Health Survey; PCS-12, Physical Component Summary of the 12-Item Short Form Health Survey; SD, standard deviation; SLEDAI-2K, Systemic Lupus Erythematosus Disease Activity Index 2000; SLICC/ACR, Systemic Lupus Erythematosus International Collaborating Clinics/American College of Rheumatology.

**Table 5:** Univariable and multivariable logistic regression analyses of factors associated with lupus retinopathy in patients with systemic lupus erythematosus (*n* = 159).

Variable	Univariable OR (95% CI)	*p*-Value	Adjusted OR (95% CI)	*p*-Value
Age at disease onset (per year)	0.97 (0.90–1.05)	0.449	—	—
Disease duration (per month)	1.00 (0.98–1.01)	0.66	—	—
Renal involvement (yes vs. no)	5.20 (1.42–19.04)	0.013	2.31 (0.56–9.58)	0.247
Mucocutaneous involvement (yes vs. no)	0.97 (0.29–3.21)	0.963	—	—
Musculoskeletal involvement (yes vs. no)	1.29 (0.39–4.23)	0.674	—	—
Hematological involvement (yes vs. no)	0.66 (0.18–2.45)	0.536	—	—
Neuropsychiatric involvement (yes vs. no)	2.37 (0.75–7.47)	0.14	—	—
Severe disease activity (SLEDAI-2K > 10)	10.08 (2.73–37.17)	<0.001	7.20 (1.78–29.11)	0.006
SLICC/ACR Damage Index (per unit increase)	1.40 (0.88–2.22)	0.152	—	—
PCS-12 score (per point increase)	0.94 (0.88–1.00)	0.044	—	—
MCS-12 score (per point increase)	0.99 (0.94–1.05)	0.826	—	—
ESR (per mm/hour increase)	1.02 (1.01–1.04)	0.005	—	—
CRP (per mg/L increase)	1.01 (1.00–1.02)	0.278	—	—
Hemoglobin (per g/dL increase)	0.75 (0.57–1.00)	0.049	—	—
Anti-dsDNA positivity (yes vs. no)	2.63 (0.56–12.35)	0.222	—	—
Low complement C3 (yes vs. no)	1.49 (0.39–5.65)	0.56	—	—
Low complement C4 (yes vs. no)	2.62 (0.87–7.90)	0.088	—	—
Antiphospholipid antibody positivity (yes vs. no)	1.96 (0.56–6.78)	0.29	—	—
Thrombocytopenia (yes vs. no)	1.32 (0.27–6.40)	0.733	—	—

**Legend:** Odds ratios (ORs) and 95% confidence intervals (CIs) were estimated using binary logistic regression analysis. Variables with *p* < 0.10 in the univariable analyses were considered for multivariable analysis; however, because only 16 patients had lupus retinopathy, we deliberately restricted the final model to two clinically relevant variables, renal involvement and severe disease activity, to minimize the risk of overfitting. We analyzed laboratory variables with missing data using available case analysis. CRP measurements were available for 70 participants (62 without retinopathy and eight with retinopathy); therefore, the CRP analysis was based on the available cases. We coded the binary variables as present versus absent. An em dash (—) indicates variables not included in the final multivariable model. **Abbreviations:** anti-dsDNA, anti-double-stranded DNA; CI, confidence interval; CRP, C-reactive protein; ESR, erythrocyte sedimentation rate; MCS-12, Mental Component Summary of the 12-Item Short Form Health Survey; OR, odds ratio; PCS-12, Physical Component Summary of the 12-Item Short Form Health Survey; SLEDAI-2K, Systemic Lupus Erythematosus Disease Activity Index 2000; SLICC/ACR, Systemic Lupus International Collaborating Clinics/American College of Rheumatology.

**Table 6:** Association between antiphospholipid antibody status and retinopathy phenotype and ocular imaging findings among patients with lupus retinopathy.

Finding	*n* (%)
**A. Retinopathy phenotype according to aPL status (*n* = 12)**	
**aPL-positive (*n* = 5)**	
Occlusive retinopathy	**3 (60.0)**
Non-occlusive retinopathy	**2 (40.0)**
**aPL-negative (*n* = 7)**	
Occlusive retinopathy	**0 (0.0)**
Non-occlusive retinopathy	**7 (100.0)**
**B. Ocular imaging findings among patients undergoing FFA and OCT (*n* = 8)**	
**Fundus fluorescein angiography findings**	
Bilateral capillary non-perfusion	8 (100.0)
Perivascular leakage	6 (75.0)
Perivascular sheathing	5 (62.5)
**Optical coherence tomography findings**	
Macular edema	8 (100.0)
Cystoid macular edema	4 (50.0)

**Legend:** Data are presented as *n* (%). For aPL analysis, percentages were calculated within the aPL-positive (*n* = 5) and aPL-negative (*n* = 7) groups. Antiphospholipid antibody (aPL) positivity was defined as positivity for one or more of the following: lupus anticoagulant (LA), anticardiolipin (aCL) IgG or IgM, and anti-β_2_-glycoprotein I (anti-β_2_GPI) IgG or IgM. We evaluated the association between aPL status and retinopathy phenotype using Fisher’s exact test (*p* = 0.045). For imaging findings, percentages were calculated among the eight patients who underwent both FFA and OCT. Patients could have more than one imaging abnormality; therefore, imaging findings were not mutually exclusive, and the percentages do not necessarily sum to 100%. The aPL analysis was exploratory because aPL testing was performed based on clinical indications and was not systematic. **Abbreviations:** aCL, anticardiolipin antibody; anti-β_2_GPI, anti-β_2_-glycoprotein I antibody; aPL, antiphospholipid antibody; FFA, fundus fluorescein angiography; LA, lupus anticoagulant; OCT, optical coherence tomography.

## Data Availability

The datasets generated and/or analyzed during the current study are available from the corresponding author upon reasonable request.

## Abbreviations

Abbreviation	Definition
aCL	Anticardiolipin
ACR	American College of Rheumatology
AI	Artificial Intelligence
anti-dsDNA	Anti-double-stranded DNA
anti-β_2_GPI	Anti-β_2_-glycoprotein I
aPL	Antiphospholipid antibody
C3	Complement component 3
C4	Complement component 4
CI	Confidence interval
CRP	C-reactive protein
EULAR	European Alliance of Associations for Rheumatology
ESR	Erythrocyte sedimentation rate
FFA	Fundus fluorescein angiography
IQR	Interquartile range
LA	Lupus anticoagulant
MCS-12	Mental Component Summary of the 12-Item Short Form Health Survey
OCT	Optical coherence tomography
OCTA	Optical coherence tomography angiography
OR	Odds ratio
PCS-12	Physical Component Summary of the 12-Item Short Form Health Survey
SD	Standard deviation
SLE	Systemic lupus erythematosus
SLEDAI-2K	Systemic Lupus Erythematosus Disease Activity Index 2000
SLICC/ACR	Systemic Lupus International Collaborating Clinics/American College of Rheumatology Damage Index
STROBE	Strengthening the Reporting of Observational Studies in Epidemiology
